# Development and validation of a multimodal predictive model based on clinical, biochemical, and quantitative dual-energy CT parameters: for predicting the benignity and malignancy of thyroid nodules

**DOI:** 10.3389/fendo.2026.1790842

**Published:** 2026-06-24

**Authors:** Yafei Zhang, Congyan Yin, Ranran Huang, Guowei Zhang, Xuhong Pan

**Affiliations:** Department of Radiology, Yantaishan Hospital, Yantai, Shandong, China

**Keywords:** dual-energy CT, logistic regression, multimodal, prediction model, thyroid nodule

## Abstract

**Objective:**

This study aimed to develop and validate a clinical prediction model integrating clinical characteristics, biochemical markers, and quantitative dual-energy CT (DECT) parameters to differentiate malignant from benign thyroid nodules.

**Methods:**

This retrospective study included 172 patients with thyroid nodules (87 malignant and 85 benign). All patients underwent non-contrast and dual-phase contrast-enhanced dual-energy CT (DECT) of the thyroid. Candidate features for model development included clinical variables (sex and age), biochemical markers (CEA, TPOAb, FT4, TSH, FT3, TRAb, Tg, CT, and TgAb), and spectral CT-derived quantitative parameters (thyroid nodule volume [TV], spectral curve slope, effective atomic number, calcium, hydroxyapatite [HAP], iodine concentration, and ICDNR). The patients were randomly divided into a training cohort and a validation cohort at a 7:3 ratio. Feature selection was performed using the least absolute shrinkage and selection operator (LASSO) and the Boruta algorithm. A predictive model was then developed and internally validated. Model performance was evaluated using the area under the receiver operating characteristic curve (AUC), calibration curves, and decision curve analysis (DCA) to assess discrimination, calibration, and clinical utility.

**Results:**

Multivariable logistic regression identified age (OR = 0.93, *p* < 0.001), TSH (OR = 1.65, *p* = 0.037), and thyroid nodule volume (TV, OR = 0.91, *p* = 0.017) as independent predictors of malignancy. HAP-MN and ICDNR were retained as auxiliary predictors following LASSO/Boruta feature selection and sensitivity analysis. The final model demonstrated good discriminative performance, with an AUC of 0.866 (95% CI: 0.802–0.930) in the training cohort and 0.852 (95% CI: 0.742–0.961) in the validation cohort. Model diagnostics indicated an acceptable fit. Goodness-of-fit tests and calibration curves showed good agreement between predicted and observed outcomes, and DCA further confirmed the model’s favorable clinical utility.

**Conclusion:**

In this study, we successfully developed a multimodal predictive model integrating clinical, biochemical, and spectral CT-derived quantitative features, which demonstrated excellent diagnostic accuracy for thyroid nodules. This non-invasive and objective tool may improve risk stratification, reduce unnecessary interventions, and support personalized patient management.

## 1 Introduction

Thyroid cancer is the most common malignant tumor of the endocrine system, and its global incidence has risen steadily in recent decades. Asia has one of the highest incidence rates worldwide (1), particularly among women of reproductive age (2). With the widespread adoption of advanced imaging techniques, many asymptomatic nodules are now detected early, with a reported prevalence as high as 76% in some populations (3). However, only 5%–10% of these nodules are malignant—predominantly papillary thyroid carcinoma (85%–90%) (4)—while the majority are benign lesions, such as nodular goiter and thyroid adenoma (5). This discrepancy has led to a significant increase in invasive surgeries for benign nodules, despite fundamental differences in the clinical management strategies required for benign versus malignant lesions (6).

Currently, fine-needle aspiration biopsy (FNAB) remains the gold standard for evaluating thyroid nodules. However, approximately 20%–30% of FNAB results are indeterminate. Although most of these nodules are ultimately confirmed as benign postoperatively, indeterminate findings often necessitate surgical resection for definitive diagnosis, posing a major challenge in clinical practice (7–9). Therefore, accurate preoperative differentiation of thyroid nodules is critically important for optimizing treatment strategies and avoiding unnecessary interventions.

Advanced imaging techniques have significantly improved the preoperative assessment of thyroid nodules. Ultrasound remains the primary imaging modality, with features such as nodule size, multifocality, extrathyroidal extension, and cervical lymph node status serving as important model features of malignancy (10, 11). More recently, dual-energy computed tomography (DECT) has emerged as a valuable tool for characterizing thyroid nodules. Its quantitative parameters—such as effective atomic number, iodine concentration, calcium deposition, and hydroxyapatite content—can serve as predictive indicators for distinguishing benign from malignant nodules, demonstrating strong diagnostic performance (12). These modalities provide rich datasets; quantitative parameters reflect tissue composition, vascular distribution, and mineralization patterns, which differ between benign and malignant tissues. Such features help identify low-risk nodules suitable for monitoring, as well as those with suspicious features (e.g., irregular margins or microcalcifications) that warrant closer evaluation (13).

The evaluation of thyroid nodules extends beyond imaging and requires the integration of clinical and biochemical information. Established risk factors include advanced age, female sex, obesity, metabolic syndrome, and estrogen dominance (14, 15). Patient demographics such as age and sex are correlated with the development and progression of thyroid nodules, with varying malignancy risks across populations (16). Furthermore, biochemical markers—including thyroglobulin (Tg), Tgantibody (TgAb), thyroid-stimulating hormone (TSH), carcinoembryonic antigen (CEA), TSH receptor antibody (TRAb), thyroid peroxidase antibody (TPOAb), free triiodothyronine (FT3), free thyroxine (FT4),and calcitonin (CT)—reflect thyroid functional status and may indicate autoimmune disorders or malignancy, thereby serving as auxiliary tools for characterization (16).

Given the multifactorial nature of thyroid nodule assessment, this study aims to develop and validate a multimodal predictive model. By integrating clinical characteristics, biochemical markers, and spectral CT quantitative parameters into a multivariable logistic regression analysis, we constructed a nomogram to provide clinicians with a robust, visually interpretable tool for the precise preoperative discrimination of benign and malignant thyroid nodules.

## 2 Materials and methods

### 2.1 Patient data

Study data were obtained from patients with thyroid nodules who were examined at Yantaishan Hospital between January 2024 and October 2025. The diagnosis was confirmed by histopathology, and a total of 172 cases were included, comprising 87 malignant and 85 benign nodules. Because this was a retrospective study that included all eligible cases during the study period, no formal *a priori* sample size calculation was performed. Based on the events-per-variable (*EPV*) principle, the sample size was considered sufficient for model development and internal validation (**Supplementary Material 1**). Clinical and biochemical variables collected for analysis included sex, age, CEA, TPOAb, FT4, TSH, FT3, TRAb, Tg, CT, and TgAb.

Inclusion criteria:

1. Adult patients with a confirmed thyroid lesion;
2. Histopathological diagnosis obtained from surgical resection or biopsy, serving as the reference standard for classification as benign or malignant;
3. Availability of complete spectral (dual-energy) CT imaging data and full biochemical and clinical records within one week prior to surgery or biopsy.

Exclusion criteria:

1. Spectral CT images of inadequate quality for quantitative analysis;
2. Prior thyroid surgery or interventional treatment that could have altered anatomy or biochemical parameters;
3. Concomitant thyroid disorders likely to confound imaging findings or laboratory assessments.

This retrospective study was approved by the Ethics Committee of Yantaishan Hospital (approval number: LL-2025-114-L). The approval covered all aspects of the study, including retrospective data collection, data analysis, and publication of results. Due to the retrospective design, the requirement for informed consent was waived by the ethics committee. All patient data were anonymized prior to analysis. The study was conducted in accordance with the Declaration of Helsinki.

### 2.2 Data collection

#### 2.2.1 Spectral CT scanning parameters

Spectral CT was performed on a GE Discovery CT750 HD scanner (GE Healthcare) using the Gemstone Spectral Imaging (GSI) protocol. Patients were scanned in the supine position with the shoulders depressed and the chin elevated, and were instructed to remain still and avoid swallowing. The scan range extended from the skull base to the level of the aortic arch in the upper mediastinum. Non-contrast and dual-phase contrast-enhanced scans (arterial and venous phases) were acquired. The acquisition parameters were as follows: rapid tube voltage switching between 140 and 80 kVp with a switching time of 0.5 ms, GSI Assist mode, automatic tube current modulation, tube rotation time of 0.8 s per rotation, pitch of 0.992, slice thickness of 1.25 mm, reconstruction interval of 0.625 mm, and noise index of 15. A non-ionic iodinated contrast agent (300 mg I/mL) was injected via an antecubital vein using a power injector at a rate of 3.0 mL/s at a dose of 1.0 mL/kg. Arterial- and venous-phase images were obtained at 35 s and 55 s after contrast injection, respectively. Unenhanced images were reconstructed using a standard algorithm. In addition, 70 keV monoenergetic images and the GSI data file were reconstructed for subsequent quantitative spectral analysis. Radiation dose parameters, including the dose-length product (DLP), were retrospectively retrieved from the scanner-generated dose reports. The effective radiation dose was calculated by multiplying the DLP by the standard neck conversion coefficient of k=0.0059 mSv/(mGy·cm).

#### 2.2.2 Spectral CT data measurement

Image data were transferred to the GE Advantage Workstation 4.7, and the spectral data file was processed using GSI Viewer 3D software to generate material density maps for effective atomic number (Zeff), calcium (Ca), hydroxyapatite (HAP), and iodine.

Two radiologists with intermediate professional titles independently performed the measurements. Regions of interest (ROIs) were manually delineated on the axial images at the maximum cross-sectional area of the thyroid nodule, and an equally sized reference ROI was placed in the contralateral normal thyroid parenchyma. A dynamic adjustment strategy was used for ROI delineation: for nodules with well-defined margins, the entire lesion was contoured whenever possible; for poorly defined nodules, more than two-thirds of the solid component was outlined while avoiding necrotic areas, cystic degeneration, coarse calcifications, and artifacts, so as to ensure measurement accuracy and consistency. For both the lesion ROI and the reference ROI, the following parameters were recorded: the mean value (Avg), relative standard deviation (Rel), maximum value (Max), and minimum value (Min) of Zeff, Ca, HAP, and iodine. In addition, CT attenuation values at 40, 100, and 140 keV monoenergetic levels were recorded. The three-dimensional dimensions of each lesion (length, width, and height) were also measured and documented. Any major discrepancy between the two radiologists was resolved by consensus.

The intraclass correlation coefficient (ICC) was calculated for all spectral CT quantitative parameters. All ICC values were >0.75, indicating good interobserver agreement and supporting the use of these quantitative parameters for subsequent statistical analyses. A schematic illustration of the measurement procedure and the calculation of spectral CT parameters is provided in the **Supplementary Materials 2**, **3**.

Accordingly, the candidate predictors for model development included: Sex, Age, CEA, TPOAb, FT4, TSH, FT3, TRAb, Tg, CT, TgAb, TV, (40–140 keV) λHu, (40–100 keV) λHu, EA, Zeff-Avg, Zeff-Max, Zeff-Min, Zeff-Rel, Zeff-MN, Ca-Avg, Ca-Max, Ca-Min, Ca-Rel, Ca-MN, HAP-Avg, HAP-Max, HAP-Min, HAP-Rel, HAP-MN, iodine-Avg, iodine-Max, iodine-Min, iodine-Rel, NICP, ICD, and ICDNR. Since there were no missing values among the variables ultimately included in the analysis, complete-case analysis was applied.

### 2.3 Statistical analysis

**Figure 1:**
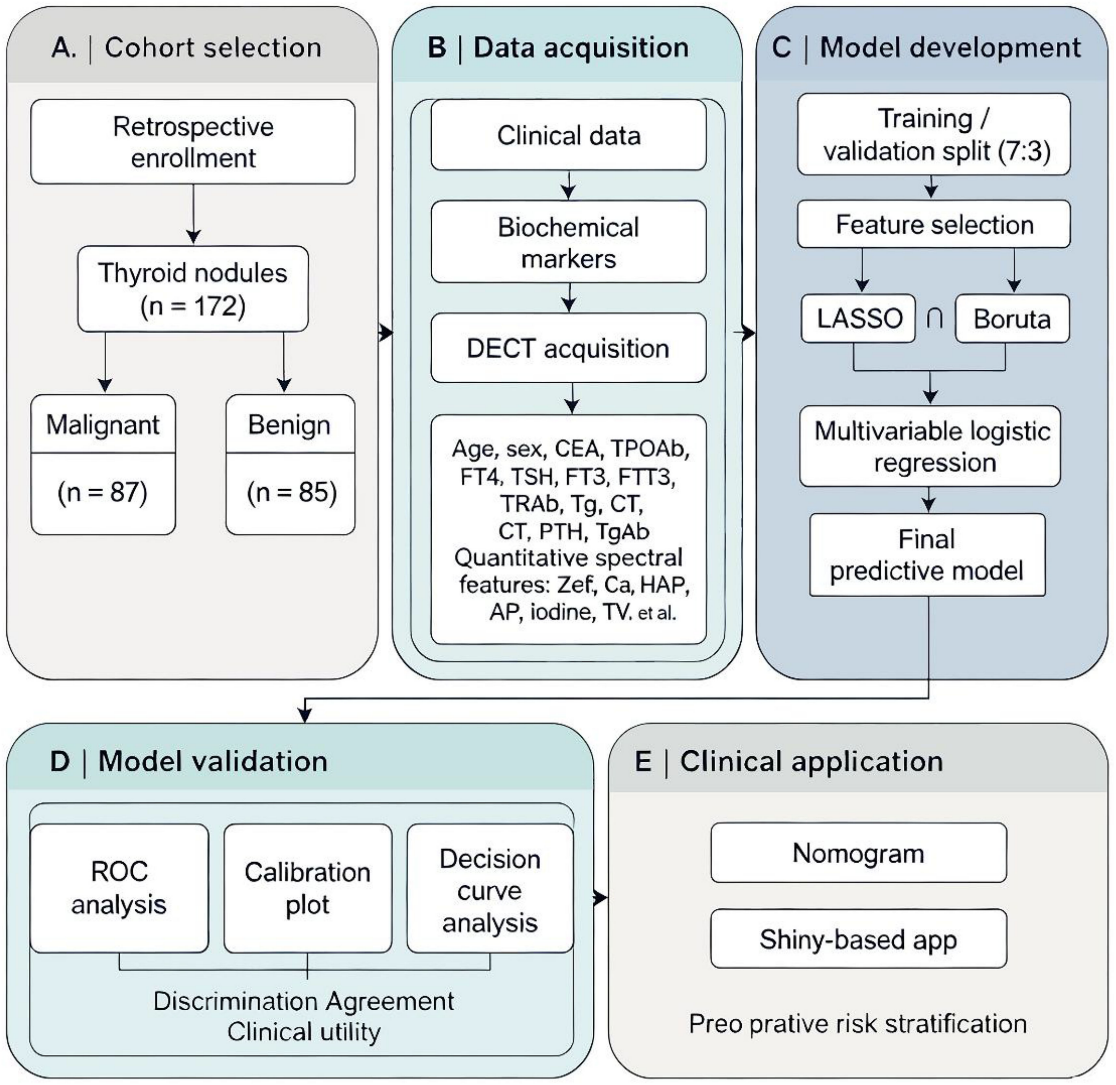
Flow diagram of the study. **(A)** Cohort selection of 172 thyroid nodule cases divided into malignant and benign cohorts. **(B)** Data acquisition, including clinical data, biochemical markers, dual-energy CT (DECT), and quantitative spectral features. **(C)** Model development, including data splitting, feature selection using LASSO and Boruta, and logistic regression analysis to build the final predictive model. **(D)** Model validation through ROC analysis, calibration plots, and decision curve analysis to evaluate discrimination, agreement, and clinical utility. **(E)** Clinical application, including the construction of a nomogram, a Shiny-based application, and preoperative risk stratification.

The dataset was randomly divided into a training cohort and a validation cohort at a 7:3 ratio, and baseline variables were compared between the two cohorts. Continuous variables are presented as median (interquartile range). In the univariate analysis, categorical variables were compared using the chi-square test, whereas continuous variables were compared using the rank-sum test. In the training cohort, variable selection was performed using the least absolute shrinkage and selection operator (LASSO) and the Boruta algorithm, and a logistic regression model was subsequently constructed. Independent predictors were identified by multivariable analysis, and sensitivity analysis was performed to assess the incremental value of candidate variables. Model diagnostics were evaluated by examining multicollinearity, the linearity assumption for continuous model features, and influential observations. Model performance was assessed using receiver operating characteristic (ROC) curves, bootstrap internal validation, and calibration curves, while decision curve analysis (DCA) was used to estimate net clinical benefit. To facilitate clinical application, an interactive dynamic prediction application was developed based on the Shiny framework. All statistical tests were two-sided, and a *p*-value < 0.05 was considered statistically significant. All statistical analyses were performed using R software (version 4.2.2). The study workflow is shown in **Figure 1**.

## 3 Results

### 3.1 Patient characteristics

A detailed comparison of clinical, biochemical, and DECT parameters between benign and malignant nodules within each cohort is presented in **Table 1**. The training cohort (n = 120) and validation cohort (n = 52) were comparable in terms of sex, age, nodule volume, most biochemical markers, and spectral CT parameters (all *p* > 0.05), with the exception of thyroglobulin (Tg), which differed significantly between groups (*p* = 0.044).

**Table 1:** Patient demographics and baseline characteristics between benign and malignant thyroid nodules.

Characteristic	Benign N = 85	Malignant N = 87	Statistic^1^	P-value
Year, Mean ± SD	57 ± 13	43 ± 12	7.29	<0.001^2^
Sex, n (%)			1.71	0.191^3^
F	67 (78.8%)	61 (70.1%)		
M	18 (21.2%)	26 (29.9%)		
CEA, Median (Q1, Q3)	1.63 (1.20, 2.19)	1.22 (0.74, 1.66)	4,893.00	<0.001^4^
TPOAb, Median (Q1, Q3)	13 (10, 15)	12 (9, 15)	3,884.00	0.567^4^
FT4, Mean ± SD	16.17 ± 3.88	16.48 ± 2.24	-0.64	0.522^2^
TSH, Median (Q1, Q3)	1.42 (0.86, 2.16)	2.42 (1.40, 3.21)	2,130.50	<0.001^4^
FT3, Median (Q1, Q3)	4.94 (4.41, 5.19)	4.81 (4.48, 5.23)	3,651.00	0.888^4^
TRAb, Median (Q1, Q3)	0.86 (0.80, 1.06)	0.91 (0.80, 1.14)	3,535.50	0.608^4^
Tg, Median (Q1, Q3)	42 (15, 95)	19 (6, 38)	4,959.00	<0.001^4^
CT, Median (Q1, Q3)	0.50 (0.50, 1.34)	0.64 (0.50, 2.00)	3,202.50	0.105^4^
TgAb, Median (Q1, Q3)	18 (16, 22)	18 (16, 57)	3,380.50	0.332^4^
TV, Median (Q1, Q3)	2 (0, 21)	1 (0, 3)	4,764.50	0.001^4^
(40-140keV)λHu, Median, Mean± SD	0.31 ± 0.28	0.31 ± 0.22	0.05	0.958^2^
(40–100 keV) λHu, Mean ± SD	0.48 ± 0.43	0.47 ± 0.35	0.10	0.919^2^
EA, Mean ± SD	69 ± 48	65 ± 29	0.55	0.583^2^
Zeff-Avg, Mean ± SD	7.80 ± 0.24	7.80 ± 0.19	0.09	0.926^2^
Zeff-Max, Median (Q1, Q3)	8.30 (8.14, 8.46)	8.26 (8.09, 8.39)	4,057.00	0.271^4^
Zeff-Min, Mean ± SD	7.21 ± 0.33	7.33 ± 0.30	-2.52	0.013^2^
Zeff-rel, Median (Q1, Q3)	0.025 (0.020, 0.030)	0.022 (0.018, 0.028)	4,266.00	0.081^4^
Zeff-MN, Mean ± SD	0.955 ± 0.038	0.943 ± 0.025	2.29	0.023^2^
calcium-Avg, Median (Q1, Q3)	3.8 (2.3, 6.6)	4.1 (2.4, 7.5)	3,541.00	0.633^4^
calcium-Max, Median (Q1, Q3)	16 (13, 21)	16 (11, 20)	4,125.00	0.191^4^
calcium-Min, Mean ± SD	-6.3 ± 5.8	-3.9 ± 4.9	-2.90	0.004^2^
calcium-rel, Median (Q1, Q3)	0.92 (0.54, 1.49)	0.78 (0.52, 1.32)	3,961.00	0.421^4^
calcium-MN, Median (Q1, Q3)	0.37 (0.19, 0.63)	0.28 (0.17, 0.46)	4,148.00	0.168^4^
HAP-Avg, Median (Q1, Q3)	8 (5, 14)	9 (5, 16)	3,499.00	0.544^4^
HAP-Max, Median (Q1, Q3)	35 (27, 44)	35 (25, 44)	4,000.00	0.355^4^
HAP-Min, Mean ± SD	-14 ± 13	-8 ± 11	-3.18	0.002^2^
HAP-rel, Median (Q1, Q3)	0.98 (0.54, 1.75)	0.79 (0.48, 1.47)	4,027.00	0.314^4^
HAP-MN, Median (Q1, Q3)	0.36 (0.19, 0.64)	0.28 (0.16, 0.47)	4,170.00	0.148^4^
iodine-Avg, Median (Q1, Q3)	2.73 (1.59, 4.63)	2.93 (1.65, 5.28)	3,595.50	0.756^4^
iodine-Max, Median (Q1, Q3)	12.0 (9.0, 14.2)	11.3 (8.0, 14.0)	4,035.00	0.302^4^
iodine-Min, Median (Q1, Q3)	-5.0 (-7.4, -2.1)	-2.4 (-5.1, 0.0)	2,594.50	<0.001^4^
iodine-rel, Median (Q1, Q3)	0.97 (0.45, 1.50)	0.85 (0.50, 1.50)	3,775.50	0.812^4^
NICP, Median (Q1, Q3)	0.35 (0.19, 0.59)	0.27 (0.16, 0.48)	4,251.00	0.090^4^
ICD, Mean ± SD	5.6 ± 4.7	7.6 ± 3.9	-2.99	0.003^2^
ICDNR, Median (Q1, Q3)	0.65 (0.41, 0.81)	0.73 (0.52, 0.84)	3,144.00	0.090^4^

1 Welch Two Sample t-test; Pearson’s Chi-squared test; Wilcoxon rank sum test; ^2^Welch Two Sample t-test; ^3^Pearson’s Chi-squared test; ^4^Wilcoxon rank sum test.

The mean dose-length product (DLP) of the study population was 917.20 ± 137.40 mGy·cm, corresponding to an estimated mean effective radiation dose of 5.41 ± 0.81 mSv.

### 3.2 Variable selection

LASSO was applied to reduce model overfitting and mitigate multicollinearity. After ten−fold cross−validation, the variables were reduced to seven potential model features: Age, TSH, TgAb, TV, Zeff-Max, HAP-MN, and ICDNR (**Supplementary Material 4**).

The Boruta algorithm was subsequently applied to identify all relevant model features, assist dimensionality reduction, and avoid prematurely discarding potentially important variables by retaining features that consistently outperform their randomized “shadow” counterparts. Boruta feature selection identified 11 important variables, 5 tentative variables, and 21 unimportant variables. The confirmed important variables were ICD, HAP-MN, HAP-Min, calcium-Min, Zeff-MN, Zeff-rel, EA, TV, Tg, TSH, Age and classified ICDNR, NICP, calcium-MN, Zeff-Min, and CEA as tentative, whereas the remaining 21 variables were deemed unimportant. Results are shown in **Supplementary Material 5**.

**Figure 2:**
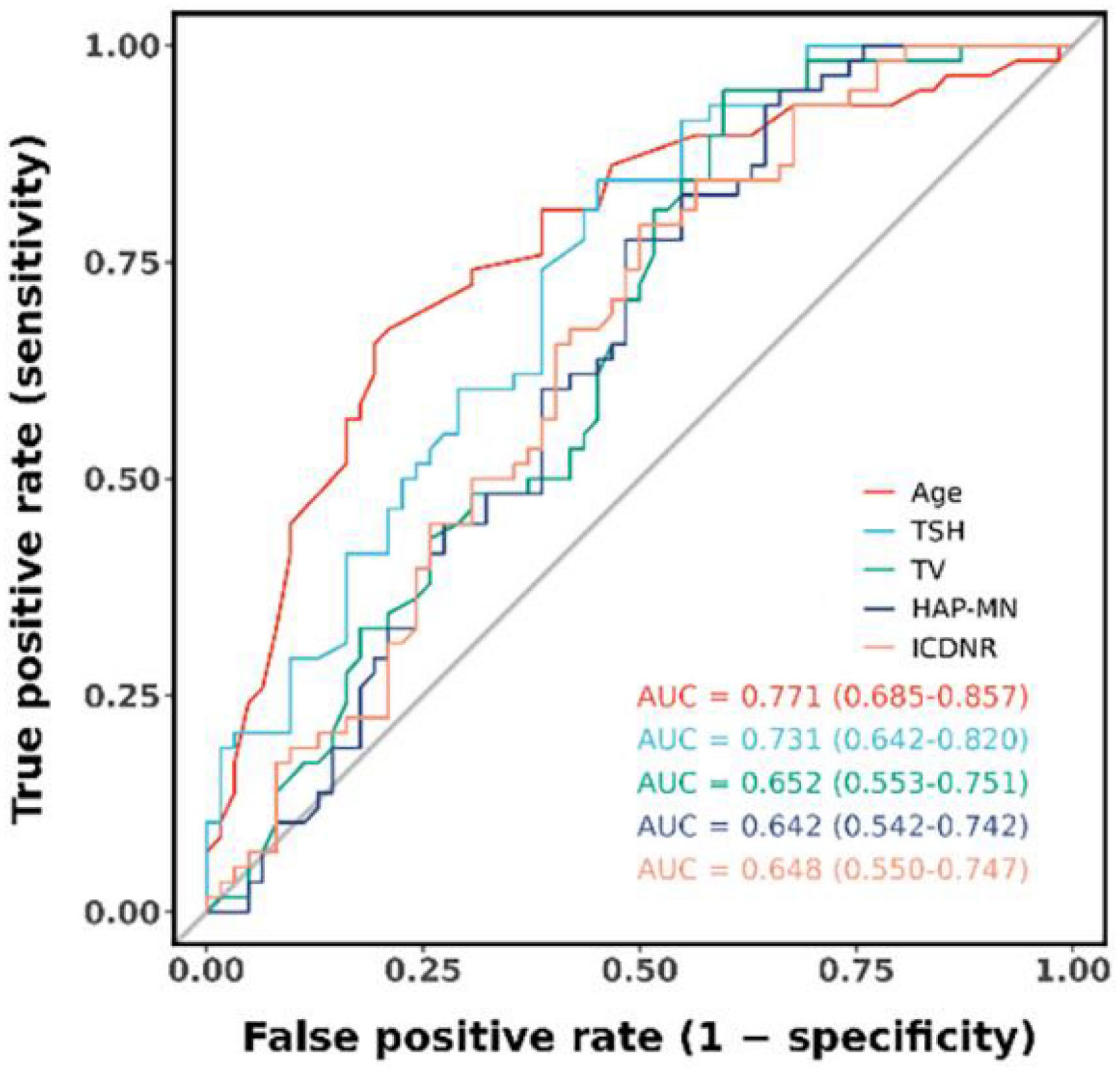
Comparison of ROC curves for individual predictive variables.

After variable selection, the intersection of model features selected by both methods was used to define the final candidate variables, yielding five model features: Age, TSH, TV, HAP-MN and ICDNR. ROC analysis of the final model features demonstrated that all AUCs exceeded 0.5 (**Figure 2**).

### 3.3 Model development

**Figure 3:**
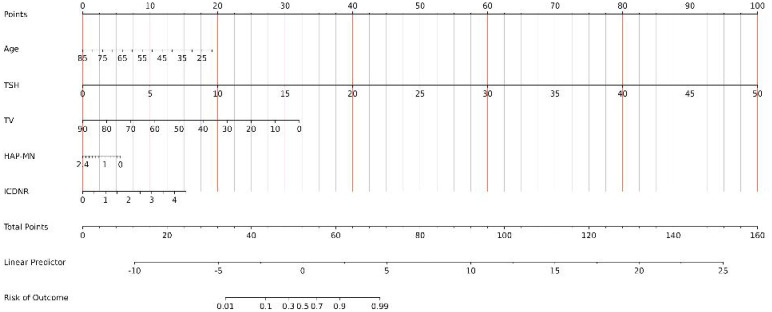
Nomogram prediction model.

The training cohort comprised 58 malignant events and five candidate predictors, corresponding to an events−per−variable (*EPV*) ratio of 11.6 (**Supplementary Material 1**), which exceeds the commonly recommended minimum of 10 and supports the feasibility of model development (17). The final multivariable logistic regression model included five model features and was translated into a user−friendly nomogram (**Figure 3**). The predictive model is accessible online at: https://zyf-thyroid.shinyapps.io/dynnomapp/.

In the training cohort, Age, TSH, and TV were identified as independent predictors, whereas HAP-MN and ICDNR did not reach statistical significance but were retained in the full model based on LASSO/Boruta feature selection (**Table 2**).

**Table 2:** Results of multivariate logistic regression for training cohort.

Characteristic	N	Event N	OR	95% CI	*P*-value
Age	120	58	0.93	0.89, 0.96	<0.001
TSH	120	58	1.65	1.09, 2.76	0.037
TV	120	58	0.91	0.84, 0.97	0.017
HAP-MN	120	58	0.56	0.07, 2.66	0.520
ICDNR	120	58	2.35	0.74, 11.14	0.203

CI, Confidence Interval; OR, Odds Ratio.

### 3.4 Model performance and validation

**Figure 4:**
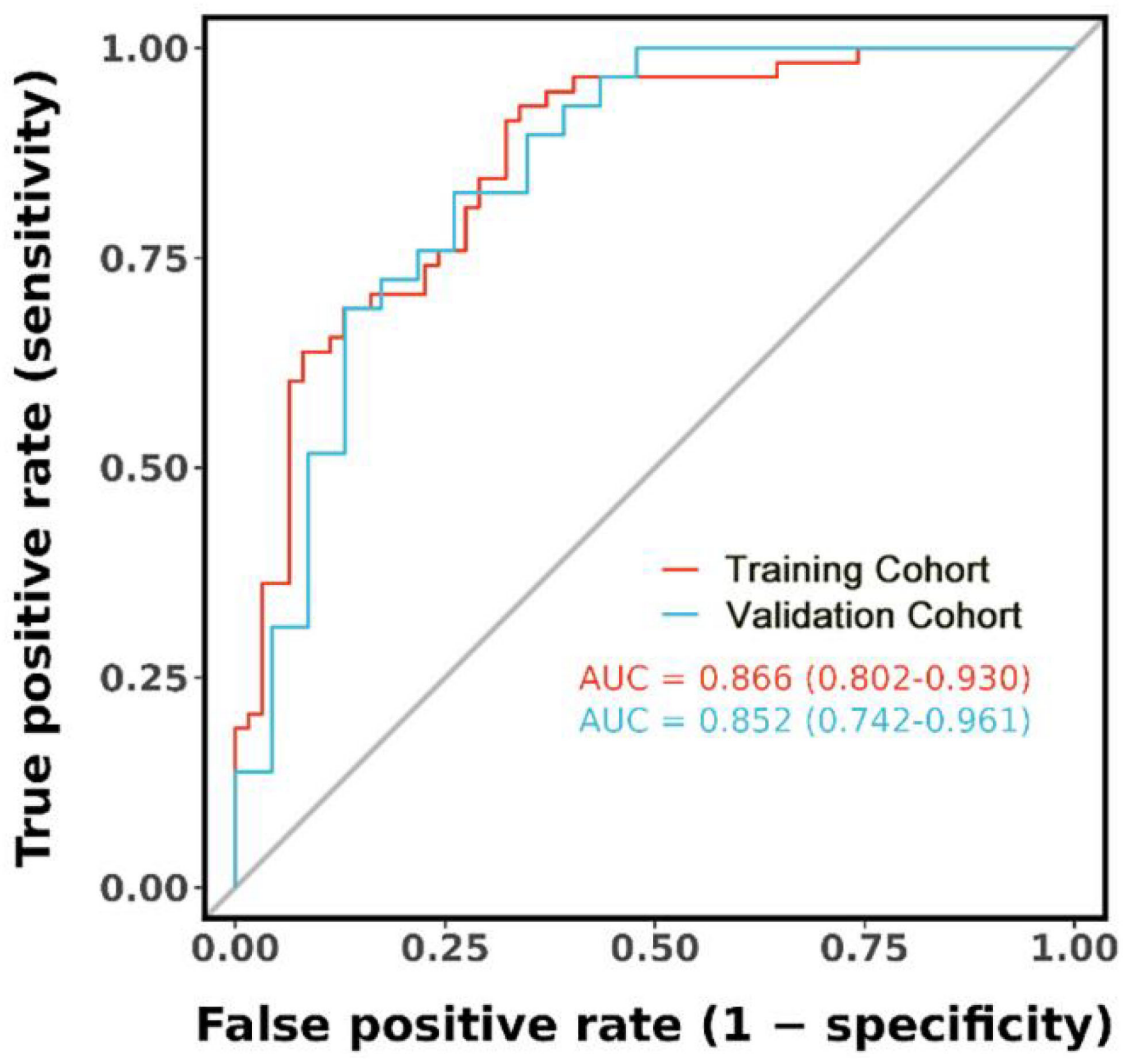
ROC curve of the prediction model in the different cohorts.

The AUC results of the model in the training and validation cohorts are shown in **Figure 4**. ROC analysis demonstrated strong discriminative performance in both cohorts. In the training cohort, the area under the ROC curve (AUC) was 0.866 (95% CI: 0.802–0.930), indicating excellent predictive accuracy. In the validation cohort, the AUC was 0.852 (95% CI: 0.742–0.961), further confirming the model’s good stability, generalizability, and robust predictive performance (**Table 3**).

**Table 3:** Performance and sensitivity analysis of the diagnostic models.

Cohort and model		AUC (95% CI)	Sensitivity (%)	Specificity (%)	PPV (%)	NPV (%)	Accuracy (%)
Training Cohort	^a^Full Model	0.866 (0.802–0.930)	75.9 (64.2–86.7)	74.2 (62.1–85.2)	73.3 (61.7–84.8)	76.7 (65.0–86.7)	75.0 (67.5–82.5)
	^b^Reduced Model	0.823 (0.762–0.884)	—	—	—	—	—
	Difference (Full vs. Reduced)	Difference: 0.0430 (95% CI: 0.0034–0.0826); DeLong *P* = 0.0334					
Validation Cohort	Full Model	0.852 (0.742–0.961)	79.3 (64.7–92.3)	73.9 (55.0–91.3)	79.3 (63.0–93.3)	73.9 (55.6–90.5)	76.9 (65.4–88.5)
	Reduced Model	0.784 (0.683–0.885)	—	—	—	—	—
	Difference (Full vs. Reduced)	Difference: 0.0680 (95% CI: 0.0009–0.1351); DeLong *P* = 0.0468					

Data in parentheses are 95% confidence intervals (CIs). The diagnostic metrics were evaluated at the default probability threshold of 0.50. For the training cohort, the Full Model demonstrated a Brier score of 0.147 and an Akaike information criterion (AIC) of 118.783. ^a^Full Model predictors: Age, TSH, TV, HAP-MN, and ICDNR. ^b^Reduced Model predictors: Age, TSH, and TV. AUC, area under the receiver operating characteristic curve; PPV, positive predictive value; NPV, negative predictive value; TV, thyroid nodule volume.

Subsequent sensitivity analysis showed that the inclusion of HAP-MN and ICDNR improved model discrimination in both the training and validation cohorts (**Table 3**).

The relatively small size of the validation cohort may have limited the precision of the performance estimates and led to wider confidence intervals. Therefore, bootstrap resampling was used in this study to estimate optimism-corrected performance and assess model stability. Bootstrap internal validation (1,000 resamples) demonstrated that the model retained good discriminative performance after optimism correction, with a corrected AUC of 0.843. The calibration slope decreased to 0.865, suggesting mild overfitting but overall acceptable calibration. The corrected Brier score was 0.166, indicating a moderate prediction error (**Supplementary Material 6**).

Model diagnostics were performed in the training cohort to assess multicollinearity, the linearity assumption of continuous model features, and the presence of influential observations. No major violations were detected (**Supplementary Materials 7**–**9**).

**Figure 5:**
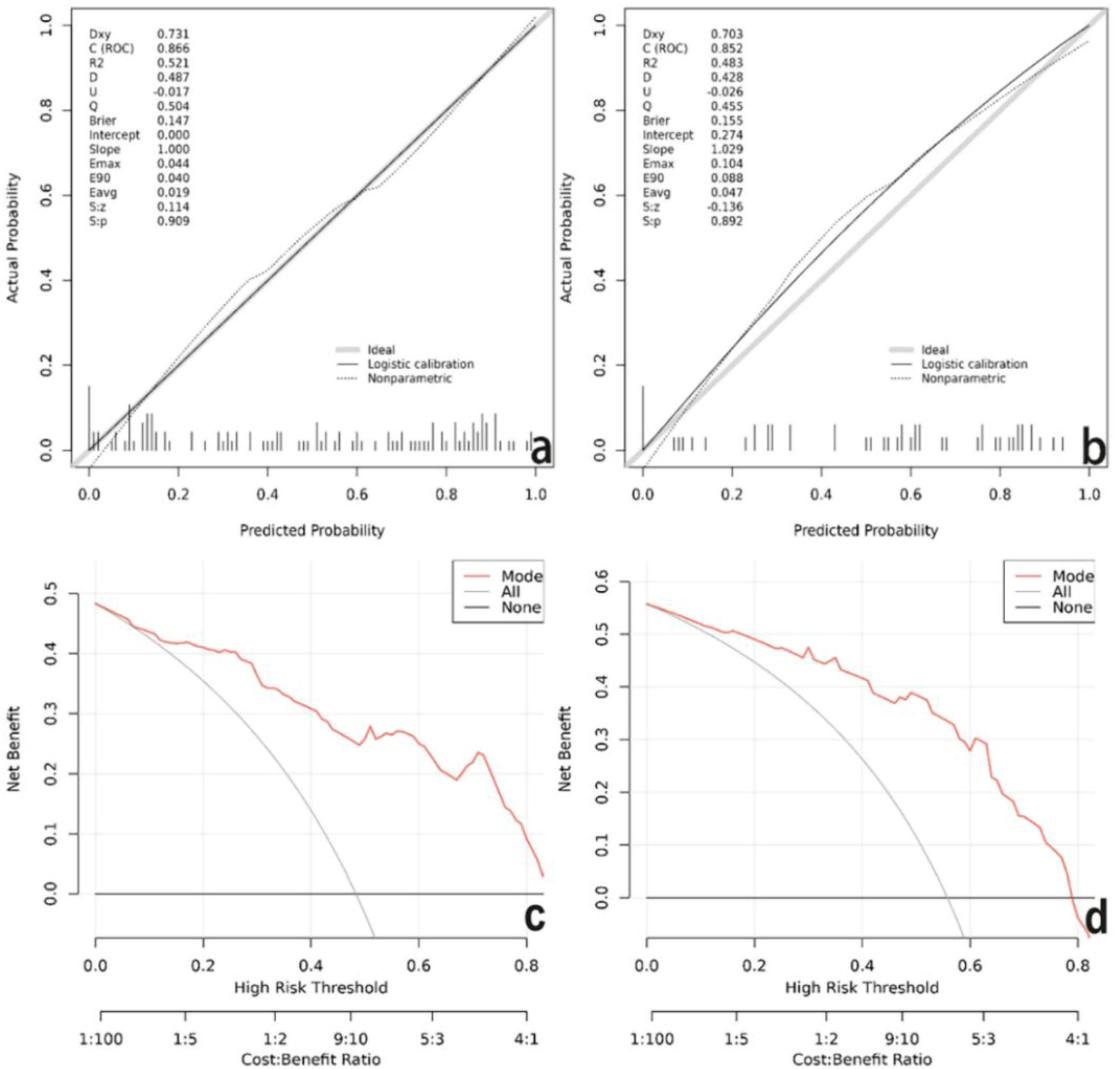
Calibration and decision curve analysis of the nomogram. **(a, b)** Calibration curves for the nomogram in the training and internal validation cohorts, demonstrating agreement between predicted and observed probabilities. **(c, d)** Decision curve analysis for the nomogram in the training and internal validation cohorts, indicating the clinical net benefit across a range of threshold probabilities.

Calibration and decision curve analysis of the nomogram are shown in **Figure 5**. The results indicate good agreement between observed outcomes and predicted probabilities. These findings confirm the validity of the original nomogram in the validation dataset, with the calibration curve closely approximating the ideal diagonal, indicating that the model’s predictions are consistent with actual observations. DCA evaluates the net clinical benefit of using the model across a range of threshold probabilities. The curves indicate that, across clinically relevant threshold probabilities, application of the nomogram provides a meaningful net benefit compared with the default strategies of treating all patients or treating none, supporting the model’s potential clinical utility.

### 3.5 Clinical implementation and decision

The predicted probability generated by the nomogram or online calculator should be interpreted as an individualized estimate of malignancy risk rather than a standalone diagnostic criterion. To support clinical implementation, we evaluated three clinically relevant thresholds in both cohorts (**Table 4**), allowing threshold selection according to clinical intent: a high-sensitivity threshold may be preferred to minimize missed malignancies in screening settings, whereas a high-specificity threshold may be more appropriate when the goal is to reduce unnecessary biopsy or surgical referral.

**Table 4:** Model performance at clinically relevant probability thresholds in the training and validation cohorts.

Threshold strategy	Threshold	Cohort	Sensitivity %,(95% CI)	Specificity %,(95% CI)	PPV %,(95% CI)	NPV %,(95% CI)	Accuracy %,(95% CI)
Youden index	0.3221	Training	93.1 (83.6–97.3)	66.1 (53.7–76.7)	72.0 (61.0–80.9)	91.1 (79.3–96.5)	79.2 (71.1–85.5)
Youden index	0.3221	Validation	89.7 (73.6–96.4)	60.9 (40.8–77.8)	74.3 (57.9–85.8)	82.4 (59.0–93.8)	76.9 (63.9–86.3)
High sensitivity	0.3347	Training	91.4 (81.4–96.3)	67.7 (55.4–78.1)	72.6 (61.4–81.5)	89.4 (77.4–95.4)	79.2 (71.1–85.5)
High sensitivity	0.3347	Validation	89.7 (73.6–96.4)	69.6 (49.1–84.4)	78.8 (62.3–89.3)	84.2 (62.4–94.5)	80.8 (68.1–89.2)
High specificity	0.6996	Training	63.8 (50.9–74.9)	91.9 (82.5–96.5)	88.1 (75.0–94.8)	73.1 (62.3–81.7)	78.3 (70.2–84.8)
High specificity	0.6996	Validation	58.6 (40.7–74.5)	100.0 (85.7–100.0)	100.0 (81.6–100.0)	65.7 (49.2–79.2)	76.9 (63.9–86.3)

Values are presented as percentages with 95% confidence intervals. PPV, positive predictive value; NPV, negative predictive value. The Youden threshold was determined in the training cohort and applied unchanged to the validation cohort.

## 4 Discussion

In this study, Age, TSH, TV, HAP−MN and ICDNR were identified as final model features derived from multimodal data. Accordingly, a predictive model integrating clinical data, biochemical markers, and spectral CT quantitative parameters was developed and validated to differentiate benign from malignant thyroid nodules. Multivariable regression analysis demonstrated that age, TSH, and TV were independent predictors. By incorporating multimodal information and applying rigorous predictor selection, the model achieved excellent discriminative performance, with AUCs of 0.866 and 0.852 in the training and validation cohorts; the model retained good discriminative performance after optimism correction, with a corrected AUC of 0.843, respectively, indicating reliable accuracy and generalizability. These results suggest that combining clinical variables, biochemical markers, and advanced imaging quantitative metrics can substantially improve the diagnostic assessment of thyroid nodules, potentially enabling timely intervention for malignant cases while reducing unnecessary invasive procedures.

In our analysis, age appeared to be a negative predictor of malignancy (OR = 0.93), suggesting that younger patients had a higher risk of malignant nodules in this cohort, which is consistent with previous epidemiological findings. Several studies have reported that papillary thyroid carcinoma in adolescents and young adults often exhibits distinct molecular and immunological features, as well as a more aggressive phenotype, including a greater propensity for lymph node metastasis and an immunosuppressive tumor microenvironment (18, 19). In contrast, other investigations have shown a higher prevalence of benign nodules among older individuals, particularly in the setting of multinodular goiter (20). These observations may help explain the age-related differences in malignancy risk; however, the relationship between age and malignancy is complex and may be influenced by tumor subtype, cohort composition, and selection bias.

TSH was associated with increased odds of malignancy (OR = 1.65), consistent with previous reports (21, 22). As a growth factor for thyroid follicular cells, TSH may promote malignant cell proliferation through TSH receptor-mediated mechanisms (15, 23). Its mitogenic effects are primarily mediated via the cAMP/PKA signaling pathway following activation of the TSH receptor, providing a plausible mechanistic basis for its potential role in thyroid carcinogenesis (24). A systematic review and meta-analysis including 23,799 subjects showed that each 1 mU/L increase in preoperative TSH was associated with a 16% increase in the risk of differentiated thyroid cancer (OR = 1.16, 95% CI: 1.06–1.27) (25). Although the precise role of TSH in thyroid carcinogenesis remains controversial, it was retained in our final model because it contributed independent predictive information in the multivariable setting. Therefore, its inclusion should be interpreted as a statistically selected predictor within a predictive framework rather than a definitive causal determinant of malignancy.

However, serum TSH levels are known to increase physiologically with age. After the age of 40 years, the upper limit of the TSH reference range rises by approximately 0.3 mIU/L per decade, suggesting an age-related shift in the set point of the pituitary–thyroid axis (26, 27). A cross-sectional analysis of 8,308 participants from the NHANES cohort also confirmed a progressive increase in the 97.5th percentile of TSH with age (28). Accordingly, the observed association between TSH and malignancy risk may be confounded by age, which is itself a recognized risk factor for thyroid cancer. Importantly, however, previous studies have suggested that the association between TSH and thyroid cancer is independent of age. Haymart et al. reported that serum TSH levels were significantly higher in patients with thyroid cancer than in those with benign disease, regardless of whether patients were older or younger than 45 years, and that higher TSH levels were independently associated with extrathyroidal extension (29). In addition, the Trivandrum prediction score, which incorporates age and TSH together with TIRADS and Bethesda categories, achieved a sensitivity of 96.2% and a specificity of 97.5% for malignancy prediction (30). Another diagnostic model incorporating age, TSH, and additional variables also confirmed the added value of TSH in improving risk stratification (31). Therefore, simultaneous inclusion of age and TSH in the predictive model may help adjust for age-related confounding, improve preoperative risk stratification of thyroid nodules, and reduce unnecessary diagnostic interventions.

Additionally, TV showed a negative association with malignancy (OR = 0.91), suggesting that larger nodules were less likely to be malignant in this cohort. Although this finding appears to contradict the traditional view that larger nodules carry greater malignant potential, it aligns with emerging evidence that very large nodules are frequently benign hyperplastic or colloid nodules (32, 33), reflecting the predominance of benign proliferative and colloid lesions (34). This apparent paradox highlights the need for comprehensive, multifactorial risk assessment rather than reliance on a single morphological feature.

Notably, the multivariable model developed in this study incorporated spectral CT-derived quantitative parameters, providing a novel approach to evaluating thyroid nodules beyond conventional clinical indicators. These quantitative measures may reflect the metabolic activity and compositional characteristics of thyroid tissue. Iodine metabolism is closely linked to thyroid function, and malignant thyroid nodules often exhibit disrupted intranodular iodine processing due to destruction of the normal follicular architecture (34, 35). Accordingly, malignant nodules generally show reduced iodine uptake, whereas some benign nodules retain functional follicular tissue and therefore exhibit relatively higher iodine uptake (12, 36). Durma et al. used dual-energy CT to detect endogenous iodine accumulation and achieve accurate diagnosis of metastatic differentiated thyroid cancer, further demonstrating marked disturbances in iodine metabolism in both primary thyroid lesions and metastatic lesions (37). Calcification is another characteristic feature of thyroid nodules and may arise through multiple mechanisms, including apoptosis, matrix mineralization, and dystrophic deposition. Among these, microcalcifications—particularly psammoma bodies—are a classic feature of papillary thyroid carcinoma (38, 39) and are mainly composed of hydroxyapatite. Spectral CT can distinguish hydroxyapatite from other calcified components through material decomposition maps. Quantitative imaging features are capable of capturing tumor heterogeneity that is not appreciable on visual inspection (11, 40, 41). Accordingly, ICDNR may quantify tissue heterogeneity related to iodine distribution, whereas the hydroxyapatite-related parameter HAP-MN may reflect the mineralized component of thyroid nodules. Although HAP-MN and ICDNR were not individually statistically significant in the multivariable analysis and both had relatively wide confidence intervals, they were retained in the model because it was designed as a multimodal predictive tool integrating clinical and imaging features rather than relying solely on conventional statistical significance. Sensitivity analysis further showed that the inclusion of HAP-MN and ICDNR improved model discrimination in both the training and validation cohorts. However, the magnitude of improvement was modest, suggesting that these spectral CT-derived variables should be regarded as auxiliary predictors rather than primary decision-makers. Their individual effects should therefore be interpreted cautiously, and their incremental diagnostic value warrants further validation in larger external cohorts.

By integrating clinical, biochemical, and spectral CT-derived features, the proposed model captures the multifactorial nature of thyroid nodule malignancy more comprehensively than approaches based on a single data source. Its discriminative performance compares favorably with several previously reported models. For instance, Song et al. developed a DSCT-radiological model with an AUC of 0.858 (42), Yuan et al. reported an AUC of 0.810 using radiomics and machine learning (43), and Liu et al. achieved an AUC of 0.864 by combining ultrasound features with hemodynamic parameters (44). Although these studies are encouraging, most rely on a single imaging modality and do not incorporate clinical or biochemical context. Ultrasound-based risk stratification systems, particularly TI-RADS and EU-TIRADS, remain the current standard for preoperative evaluation of thyroid nodules. In a systematic review and meta-analysis of 21,882 nodules, ACR TI-RADS achieved a pooled sensitivity of 0.89, specificity of 0.70, and summary AUC of 0.86 (45). For EU-TIRADS, previous studies have reported a pooled unnecessary biopsy rate of 38% (46). In the present study, our multimodal model achieved an AUC of 0.866, with a sensitivity of 0.759 and a specificity of 0.742, suggesting performance that is at least comparable to established ultrasound-based systems. Notably, the model incorporates TSH and spectral CT-derived parameters such as HAP-MN and ICDNR, which may provide functional and compositional information not directly captured by conventional sonographic assessment. These findings support the potential role of the model as a complementary tool in selected patients, particularly when sonographic findings are indeterminate or clinical and imaging features are discordant. However, any comparison with TI-RADS or EU-TIRADS should be interpreted cautiously because it is indirect, and no contemporaneous head-to-head validation was performed. Moreover, because DECT involves ionizing radiation, the model is unlikely to replace ultrasound as a first-line screening tool. Rather, it may be more appropriately positioned as a second-line modality for problem-solving in carefully selected cases, where additional preoperative information could meaningfully influence management.

This study has important clinical implications. Although thyroid nodules are highly prevalent, the proportion of malignant cases remains relatively low (4, 5). In routine practice, ultrasound- and cytology-based diagnostic pathways may still lead to unnecessary biopsies and surgeries. A validated, accessible, and accurate predictive model could therefore help optimize clinical decision-making. By integrating readily available clinical variables, biochemical markers, and spectral CT-derived quantitative parameters, our model provides a non-invasive tool that may support risk-adapted management—reducing unnecessary intervention in low-risk patients while facilitating timely treatment in high-risk cases. Although the model was developed for diagnostic discrimination rather than prognostic prediction, improved preoperative risk stratification may still have meaningful downstream effects, including fewer unnecessary surgeries, faster definitive diagnosis, more appropriate referral, and improved resource utilization.

Importantly, diagnostic performance alone does not guarantee clinical utility. The impact of the model on patient-important outcomes remains to be established. Future prospective studies should therefore assess endpoints such as unnecessary surgery rates, time to diagnosis, patient anxiety, cost-effectiveness, and recurrence-free survival to determine whether the model translates into measurable clinical benefit. In addition, the use of logistic regression and a nomogram provides greater interpretability than “black-box” machine-learning approaches, which may facilitate clinical implementation.

However, several factors may affect model transportability. Differences in patient demographics, scanner vendors, acquisition protocols, and inter-institutional imaging workflows may influence model stability. Accordingly, external validation is an essential next step before clinical adoption. Although the model performed well on a single scanner platform, extension to multicenter settings will require careful consideration of cross-platform variability. Spectral CT-derived quantitative parameters, such as iodine concentration and monochromatic CT values, can vary substantially across scanners because of differences in spectral separation, tube voltage combinations, detector technology, and post-processing algorithms. Without calibration, between-platform variability may be considerable, particularly at low iodine concentrations; phantom-based cross-calibration can substantially reduce this variation. To facilitate multicenter implementation, standardized acquisition protocols, routine quality control with multi-energy phantoms, cross-platform calibration using anthropomorphic phantoms, and normalization to internal reference structures may all be valuable strategies. Until such validation is available, the model coefficients should be regarded as scanner- and protocol-specific.

Several potential failure modes should also be acknowledged. Owing to the retrospective design and the limited number of misclassified cases, detailed re-review of false-positive and false-negative nodules was not feasible. These misclassifications likely reflect the intrinsic heterogeneity of thyroid nodules and the limited informativeness of some clinical and imaging scenarios. Accordingly, the model may be less reliable in very small nodules, predominantly cystic nodules, or nodules associated with thyroiditis. As such, it should be used as an adjunct to, rather than a replacement for, standard clinical assessment.

## 5 Limitations

Several limitations should be acknowledged. First, the retrospective single-center design may have introduced selection and information biases. Because the cohort was derived from a tertiary referral center rather than an unselected population of patients with thyroid nodules, it likely overrepresents clinically suspicious cases and a relatively high prevalence of malignancy, which may limit representativeness and introduce spectrum bias. Consequently, the model may perform differently in primary care or community-based settings. Second, the overall sample size was modest, and the validation cohort was particularly small, which may have reduced the precision of performance estimates, widened confidence intervals, and increased susceptibility to influential observations, thereby affecting the stability of some coefficient estimates. Although internal validation was encouraging, the absence of external validation means that generalizability across institutions, scanner platforms, imaging protocols, and disease-prevalence settings remains uncertain. Third, the model was not directly compared with established ultrasound-based risk stratification systems such as TI-RADS or EU-TIRADS; therefore, its incremental value over current standard-of-care pathways remains to be demonstrated. Fourth, the model was developed for diagnostic discrimination rather than prognostic prediction, and its impact on patient-centered outcomes such as unnecessary surgery, time to diagnosis, cost-effectiveness, and recurrence-free survival remains unknown. In addition, although age and TSH were simultaneously included in the multivariable model, the observed association between TSH and malignancy may still be subject to residual age-related confounding. Several informative variables, including detailed ultrasound features and molecular biomarkers such as BRAF mutation status, were not incorporated; future models integrating these factors may further improve diagnostic performance. Finally, this study was limited to logistic regression modeling, and alternative machine-learning approaches were not evaluated. Prospective multicenter studies with external validation, direct comparison against established ultrasound systems, and assessment of patient-centered outcomes will therefore be necessary before routine clinical implementation.

## 6 Conclusion

In conclusion, this study developed and internally validated a multimodal predictive model integrating clinical, biochemical, and quantitative spectral CT parameters for differentiating benign from malignant thyroid nodules. The model demonstrated good discriminatory performance and may help improve diagnostic accuracy, reduce unnecessary surgery, and support clinical decision-making. Further multicenter prospective studies are needed to validate and refine this model for precision management of thyroid nodules. Importantly, the model should not be used for clinical decision-making until its performance is confirmed in external validation cohorts.

## Data Availability

The original contributions presented in the study are included in the article/**Supplementary Material**. Further inquiries can be directed to the corresponding author.
