# Supplementary material for: Development and validation of a multimodal predictive model based on clinical, biochemical, and quantitative dual-energy CT parameters: for predicting the benignity and malignancy of thyroid nodules
Source: Front Endocrinol (Lausanne). 2026 Jun 24;17:1790842. doi: 10.3389/fendo.2026.1790842 (PMC13341447; doi:10.3389/fendo.2026.1790842)
Supplement: Supplementary file 1 [file DataSheet1.docx]

**1.** **Rationality of sample size**

A total of 5 candidate variables were included:

• Training set sample size: 120.

• Number of malignant events: 58.

• Number of candidate variables: 5.

*EPV* = 58/5 = 11.6（>10）, indicating that the sample size satisfies the requirements for logistic regression analysis.**￼**

***
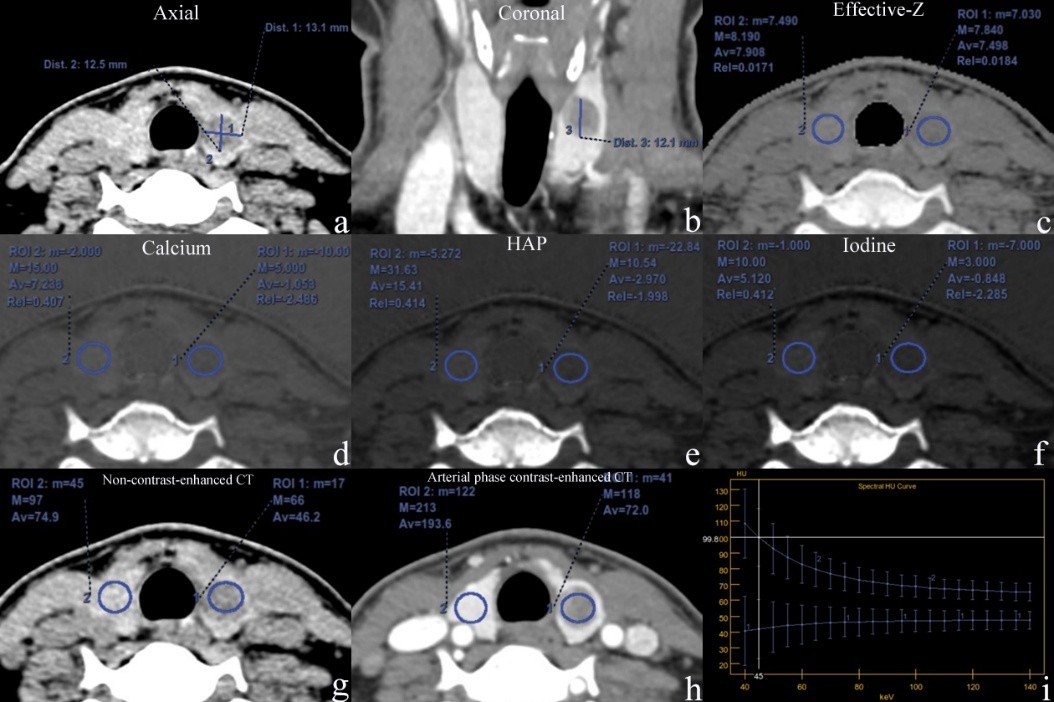
*2. Schematic diagram of quantitative parameter measurement based on spectral CT**

Figure 1. Schematic illustration of the measurement of quantitative parameters derived from spectral CT.

**3. The DECT-derived parameters were calculated as follows:**

-Mean normalization (MN) for Zeff, Ca and HAP: MN = (Avg value of the lesion side) / (Avg value of the contralateral normal side).

-Iodine concentration difference (ICD): ICD = (Avg iodine concentration of contralateral normal tissue) − (Avg iodine concentration of lesion).

-Iodine concentration difference normalized ratio (ICDNR): ICDNR = ICD / (Avg iodine concentration of contralateral normal tissue).

-Normalized iodine concentration proportion (NICP): NICP = (Avg iodine concentration of lesion) / (Avg iodine concentration of contralateral normal tissue).

-Tumor volume (TV): three‑dimensional lesion volume calculated from volumetric CT images (mL).

-Spectral curve slope (40–100 keV): λHU(40–100) = [CT(40 keV) − CT(100 keV)] / (100 − 40) (HU/keV).

-Spectral curve slope (40–140 keV): λHU(40–140) = [CT(40 keV) − CT(140 keV)] / (140 − 40) (HU/keV).

-Enhancement amplitude (EA):EA = arterial‑phase CT attenuation (lesion) − non‑contrast CT attenuation (lesion) (HU).

All above parameters and units (where applicable) were calculated and recorded for subsequent analysis.

**
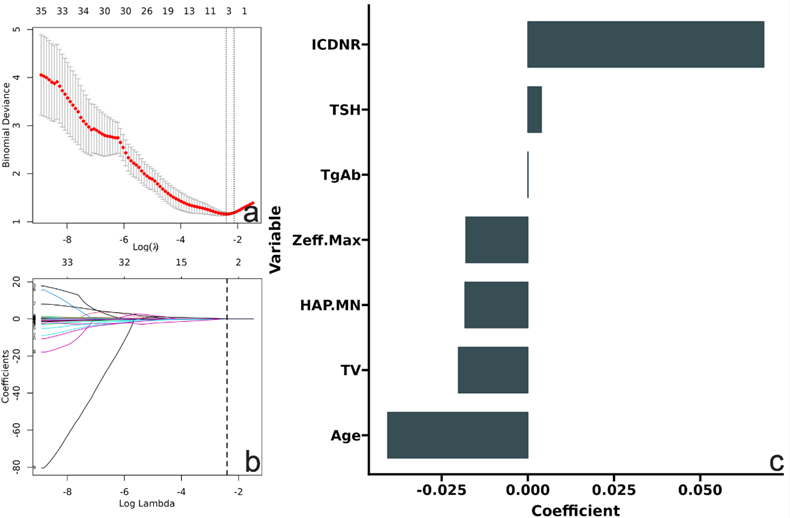
4.** **LASSO-based feature selection and predictor shrinkage**

Figure 2. LASSO-based feature selection and predictor shrinkage

(a) Ten-fold cross-validation identified the optimal tuning parameter λ (λ = 0.0905).

(b) Coefficient trajectories of candidate model features across different λ values.

(c) Variables with non-zero coefficients retained in the final LASSO model.


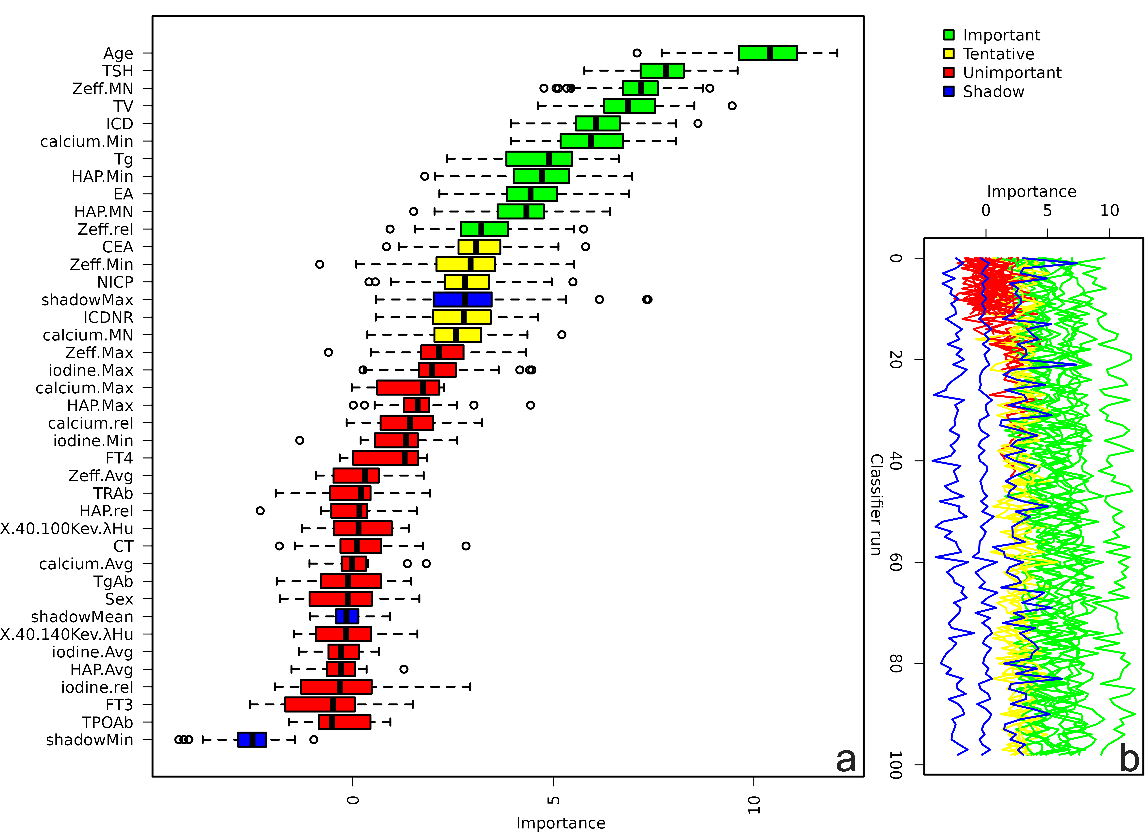
**5.** **Boruta-based feature ranking and selection**

Figure 3. Boruta-based feature ranking and selection

(a) Ranking of candidate model features by the Boruta algorithm. Important features are shown in green, tentative features in yellow, unimportant features in red, and shadow features in blue.(b) History plot of feature decisions in the Boruta procedure based on Random Forest. Confirmed attributes showed significantly higher importance than rejected attributes.

**
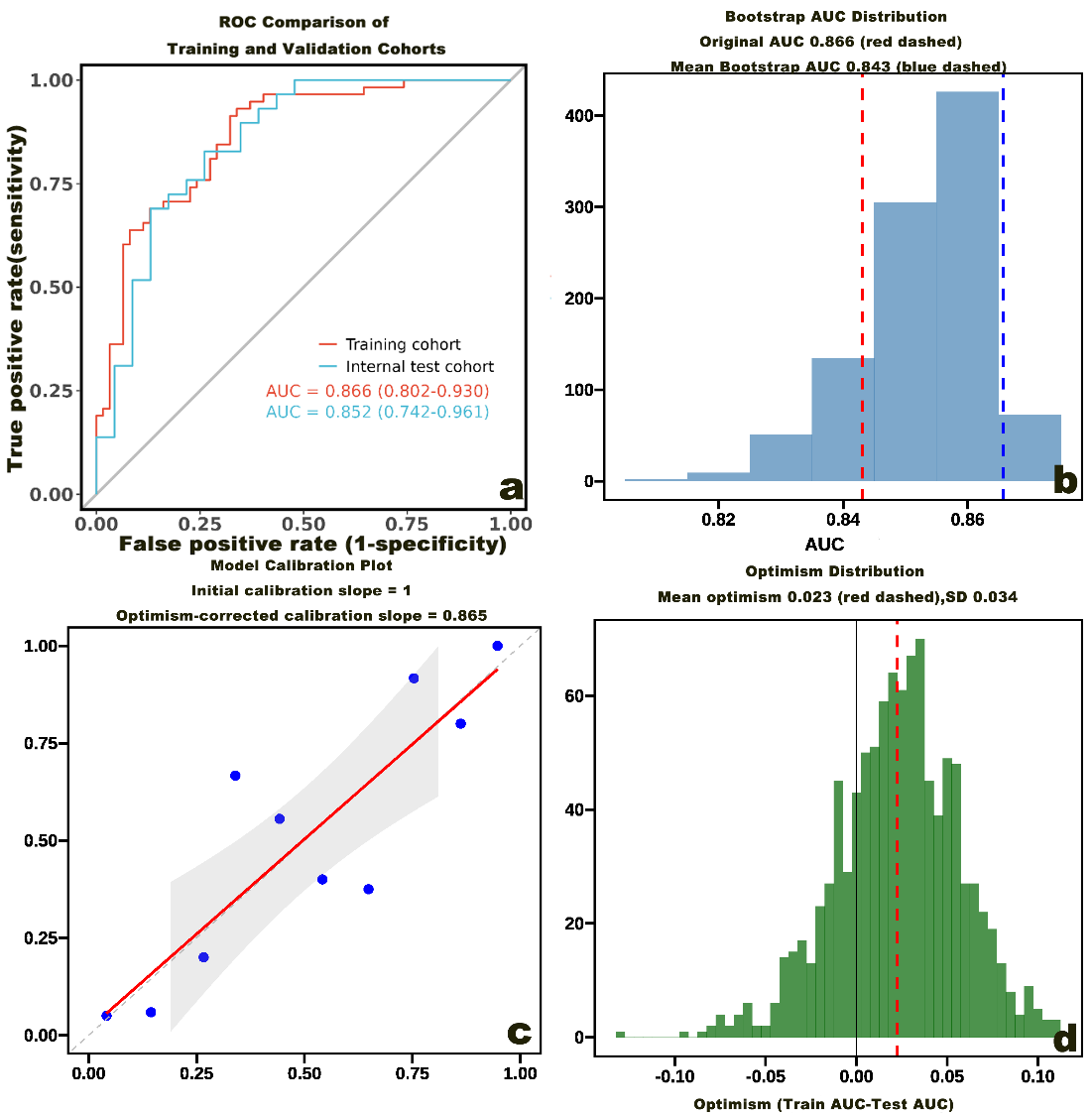
6.** **ROC comparison and bootstrap validation**

Figure 6. ROC comparison and bootstrap validation

(a) ROC curves comparing the training cohort (AUC = 0.866) and the validation cohort (AUC = 0.852). (b) Bootstrap-corrected area under the curve (AUC = 0.843).（c) Bootstrap-corrected calibration slope (0.865). (d) Mean optimism (0.023).

**7. Multicollinearity Test**

| Results of Collinearity Test | | | |
| --- | --- | --- | --- |
| Variable | VIF | Collinearity Status | Tolerance |
| Age | 1.065 | Acceptable | 0.938 |
| TSH | 1.019 | Acceptable | 0.981 |
| TV | 1.021 | Acceptable | 0.978 |
| HAP-MN | 1.179 | Acceptable | 0.847 |
| ICDNR | 1.129 | Acceptable | 0.885 |

The multicollinearity test showed that all variables had VIF values ranging from 1.019 to 1.179 and tolerance values from 0.847 to 0.981, indicating no significant multicollinearity. All variables were acceptable for inclusion in the regression model.

**8. Evaluation results from quartile trend analysis and quadratic term test**

Quartile trend analysis and quadratic term test were performed to evaluate the linear and non-linear associations between the included continuous variables and the study outcome, respectively. A two-sided *p-*value < 0.05 was considered statistically significant.

| Results of Quartile Trend Analysis and Quadratic Term Test | | |
| --- | --- | --- |
| Variable | Quartile trend test *p-*value | Quadratic term *p-*value |
| Age | <0.001 | 0.550 |
| TSH | <0.001 | 0.081 |
| TV | 0.003 | 0.996 |
| HAP-MN | 0.016 | 0.057 |
| ICDNR | 0.024 | 0.095 |

The results of quartile trend analysis showed that all included variables (Age, TSH, TV, HAP-MN, ICDNR) had statistically significant linear trends with the study outcome (all *P* for trend < 0.05). The quadratic term test revealed no significant non-linear associations between the variables and the outcome (all *P* > 0.05), except for HAP-MN showed a trend toward statistical significance (*P* = 0.057). Therefore, all variables were included in the final regression model as linear terms without additional quadratic transformation.

**9. Logistic regression model outlier diagnosis**

Outlier diagnosis of the logistic regression model was performed using Cook's distance, leverage value, and DFBETA statistics to identify influential observations that might affect model stability.

| Summary of Outlier Diagnostic Tests for Logistic Regression Model | | | | | |
| --- | --- | --- | --- | --- | --- |
| Test Method | Core Index | Test Threshold | Number of Abnormal Samples | Proportion of Abnormal Samples | Test Conclusion |
| Cook's Distance Test | Cook's Distance | 4/n = 0.0333 | 6 | 5.0% | Only 5.0% of the samples are influential points, no extreme strong influential points, and the model is robust overall. |
| Leverage Value Test | Leverage Value | 2p/n = 0.0833 | 10 | 8.3% | Only 8.3% of the samples are high-leverage points, no abnormal eigenvalues, and the results are within the acceptable range. |
| DFBETA Coefficient Influence Test | DFBETA | 2/√n = 0.1826 | 13 | 10.8% | Some samples have a slight impact on the intercept and individual coefficients, no samples with extreme impact. |

The outlier diagnosis of the logistic regression model identified 6 influential observations (5.0%) with Cook's distance exceeding the threshold of 4/n, and 10 high-leverage points (8.3%) with leverage value exceeding 2p/n. No extreme outliers were found, and the model results were confirmed to be robust by sensitivity analysis.
